# Three-Dimensional Holographic Electromagnetic Imaging for Accessing Brain Stroke

**DOI:** 10.3390/s18113852

**Published:** 2018-11-09

**Authors:** Lulu Wang

**Affiliations:** 1 Department of Biomedical Engineering, School of Instrument Science and Opto-Electronics Engineering, Hefei University of Technology, Hefei 230009, China; luluwang2015@hfut.edu.cn or lwang381@hotmail.com; Tel.: +86-18355172182; 2 Institute of Biomedical Technologies, Auckland University of Technology, Auckland 1001, New Zealand

**Keywords:** electromagnetic induction imaging, magnetic induction tomography, sensor array, brain stroke, dielectric properties

## Abstract

The authors recently developed a two-dimensional (2D) holographic electromagnetic induction imaging (HEI) for biomedical imaging applications. However, this method was unable to detect small inclusions accurately. For example, only one of two inclusions can be detected in the reconstructed image if the two inclusions were located at the same XY plane but in different Z-directions. This paper provides a theoretical framework of three-dimensional (3D) HEI to accurately and effectively detect inclusions embedded in a biological object. A numerical system, including a realistic head phantom, a 16-element excitation sensor array, a 16-element receiving sensor array, and image processing model has been developed to evaluate the effectiveness of the proposed method for detecting small stroke. The achieved 3D HEI images have been compared with 2D HEI images. Simulation results show that the 3D HEI method can accurately and effectively identify small inclusions even when two inclusions are located at the same XY plane but in different Z-directions. This preliminary study shows that the proposed method has the potential to develop a useful imaging tool for the diagnosis of neurological diseases and injuries in the future.

## 1. Introduction

Medical imaging plays an essential role in the diagnosis of malignant tumors. Early diagnosis of the malignant tumor could significantly improve the treatment outcome and prognosis [1]. X-ray, ultrasound, computed tomography (CT), magnetic resonance imaging (MRI), and positron emission tomography (PET) are the commonly used biomedical imaging tools in hospitals. However, these techniques have some limitations. X-ray produces harmful radiation to the human body [2], and it is difficult for early abnormal tissue detection due to the relatively small contrast between the healthy tissue and the abnormal tissue at X-ray frequencies. PET is an excellent choice for imaging soft tissues, but it suffers from poor image resolution and relatively high-cost [3]. CT and MRI are well-established methods for identifying structural alterations of the biological tissue, such as brain tissue. However, they are unsuitable for continuously monitoring brain disease because of the relatively high-cost and time-consuming [4,5]. CT also produces harmful ionizing radiations to the human body. It is urgently needed to develop a new imaging method for biomedical imaging and diagnostic applications especially for early diagnosis of critical diseases, such as lung cancer, brain diseases.

Electromagnetic imaging (EMI) has been proposed as a potential technique to overcome the limitations of existing biomedical imaging tools, which has received increasing attention for applications in biomedical imaging and diagnostics. In the past three decades, several research groups have investigated EMI for imaging the electromagnetic properties (EPs, conductivity, permittivity, and permeability) of biological tissues using low-frequency (<10MHz) electromagnetic (EM) spectrum [6,7,8,9]. EMI has been applied to detect several diseases, including gastric emptying [10], lung ventilation [11], breast cancer [12], prostate cancer [13], pulmonary perfusion [14], and acute cerebral stroke [15]. EMI has been extensively studied for monitoring neurological diseases and injuries, including hemorrhagic stroke [16], ischemic stroke [17], and brain edema [18]. The applicability of EMI for monitoring brain diseases relies on the significant change in the EPs of brain tissues when a stroke occurs. 

Electrical impedance tomography (EIT) is a noninvasive imaging tool which employs an array of electrodes on the surface of the biological object and maps the internal conductivity distribution changes of the object based on physiological and pathological activities [19,20]. However, the EIT technique requires expensive computational cost, may cause skin affections because of the use of the skin touch electrodes, and low spatial resolution. In 1993, Al-Zeibak et al. first reported the magnetic induction tomography (MIT) for biomedical applications [21]. An MIT system usually employs an excitation coil to induce eddy currents in the biological tissue and uses a detection coil to measure scattering field from the object to reconstruct a 2D image by using filtered back-projection. Compared to EIT, MIT is a contactless method which does not fill galvanic coupling in the space between the MIT device and the object. Therefore, the ill-defined electrode-skin interface is avoided. MIT is a more sensitive technique as it can monitor all three EPs parameters, and it is particularly attractive for identifying brain diseases (e.g., brain edema) because of the induced magnetic field via coils can penetrate through the skull much more easily than the injected currents via electrodes in the EIT system [22,23,24,25,26,27,28]. However, the MIT technique also has some drawbacks include limited image resolution, produce artifacts and the leak of clinical suitable measurement instruments. Recently, the authors developed a holographic electromagnetic induction (HEI) imaging for dielectric object imaging [29,30]. However, this method is unable to produce 3D images and unable to detect small lesions embedded in a 3D object accurately.

This paper presents the development of a 3D HEI method and investigates the feasibility and capability of the proposed method for detecting small inclusions embedded in a 3D dielectric object. A numerical system, including a realistic 3D head phantom and data processing model, has been developed to validate the effectiveness of the method for imaging infarcts that mimic a stroke embedded in the head phantom. This paper presents the theoretical model, together with simulation experiments and simulation results. 

The present work is organized as follows: Section 2 presents the principle of 3D HEI imaging concept. Section 3 describes a numerical system for validation of the method. Section 4 presents simulation results. Finally, Section 5 concludes this paper. 

## 2. Basic Principle

### 2.1. Concept of the 3D HEI System

**Figure 1:**
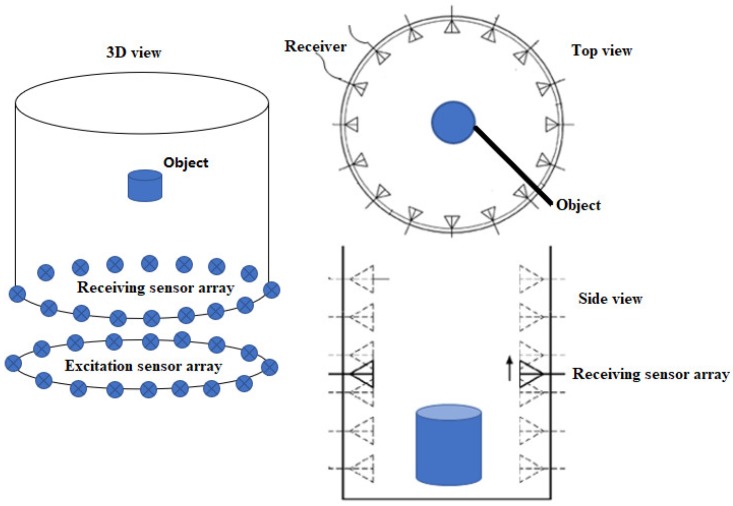
Schematic of the 3D HEI system.

Figure 1 shows a conceptual scheme of the 3D HEI system. The system consists of a cylindrical tank, a biological object, a radio frequency (RF) generator to produce EM signals, 16 excitation sensors to induce a magnetic field into the object, 16 receiving sensors to measure the scattering field from the object, a host computer with HEI program. All excitation sensors are equally arranged in a circle plane, and all receiving sensors are equally arranged in a circle plane. The excitation sensor array is located at the bottom of the tank, and the receiving sensor array plane is mounted on the wall of the tank and is designed moveable in the Z-direction. The biological object is located at the center of a cylinder (x = 0 mm, y = 0 mm, z = 0 mm). A matching medium is filled in the imaging chamber, which allows for an optimal sensor matching and ensures EM signals are propagating through the object and the scattering field can be more accurately recorded. For simplicity, a single frequency is selected as the working frequency according to previous studies [26,29]. 

We first place the receiving sensor array at one of the H vertical locations, the RF generator produces EM signals to excitation sensors, let one of the excitation sensors creates a magnetic field which leads to an electric field that drives an eddy current in the object, and every receiving sensor measures the backscattered signals from the object. This process is repeated until all excitation sensors have created a magnetic field. A 2D HEI image is obtained over the cross-section of the 3D object on the XY plane using 2D HEI method [29]. Then, we reposition the receiving array plane at a new vertical location and repeat the image data collection process until all vertical locations have been scanned. Vertical scans in the Z-direction can provide a stack of 2D images. A 3D object image can be reconstructed by combining a stack of 2D images. 

### 2.2. Forward Model

This section presents the forward model that has been applied in the simulation experiments. The time-harmonic vector wave expression for the electric field can be solved by:

(1) $$\nabla \times \left(\mu_{r}^{-1}\nabla \times \vec{E}\right)-k_{0}^{2}\varepsilon_{r}\vec{E}=-j\omega \mu_{0}\vec{J_{s}}$$

where ∇ is the divergence operator, the relative permeability $\mu_{r}=\mu /\mu_{0}$, the complex relative permittivity $\varepsilon_{r}=\frac{\varepsilon }{\varepsilon_{0}}+\frac{j\sigma }{\varepsilon_{0}\omega }=\varepsilon^{\prime }+j\varepsilon^{\prime \prime }$, $\mu_{0}$ and $\varepsilon_{0}$ are the permeability and permittivity of free space, respectively. σ is the electrical conductivity, ω=2πf, and $k_{0}=\omega \sqrt{\mu_{0}\varepsilon_{0}}$, $\vec{E}$ denotes the electric filed, $\vec{E}={\vec{E}}_{inc}+{\vec{E}}_{scat}$ with ${\vec{E}}_{inc}$ and ${\vec{E}}_{scat}$ are the transmitted and received electric field in the sensor, $j=\sqrt{-1}$, and $\vec{J_{s}}$ is the excitation current density in the excitation coil. 

The magnetic field strength is computed using the results of the electric field $\vec{E}$ in Equation (1):

(2) $$\vec{H}=j{\left(\omega \mu_{0}\mu_{r}\right)}^{-1}\cdot \left(\nabla \times \vec{E}\right)$$

where $\vec{H}={\vec{H}}_{inc}+{\vec{H}}_{scat}$, ${\vec{H}}_{inc}$ and ${\vec{H}}_{scat}$ are the transmitted field and received magnetic field in the coil, respectively. 

### 2.3. Backward Model

**Figure 2:**
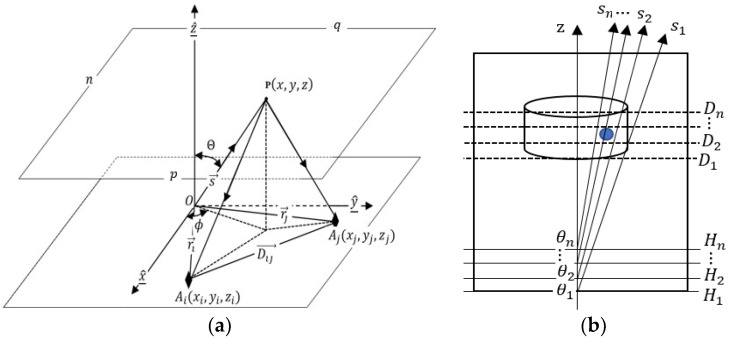
(**a**) A sensor-pair; (**b**) 3D view of sensor array plane.

We assume a target point P embedded in a 3D object (see Figure 2). The sensor array is placed at one vertical location, the scattering signals from the object can be measured by any sensor located on the sensor array plane as [30]:

(3) $${\vec{H}}_{scat}\left(\vec{r_{0}}\right)=\frac{-j}{4\pi \omega \mu_{0}}\underset{V}{\int }[\left(\vec{J_{m}}\cdot \nabla \right)\times \nabla +k_{0}^{2}\vec{J_{m}}+j\omega \mu_{0}\vec{J_{s}}\nabla ]G\left(\vec{r},\vec{r_{0}}\right)dV$$

where $\vec{r_{0}}$ is the position vector from the origin to the point source, $\vec{r}$ is the position vector from the detector to point source, G denotes Green’s function. The induced electric current density and magnetic current density can be computed by:

(4) $$\vec{J_{s}}=j\omega \varepsilon_{0}\left(\varepsilon_{r}-1\right)\vec{E};\;\vec{J_{m}}=j\omega \mu_{0}\left(\mu_{r}-1\right)\vec{H};$$

Equation (3) can be simplified as:

(5) $${\vec{H}}_{scat}\left(\vec{r_{0}}\right)=\frac{k_{0}^{2}}{4\pi }\underset{V}{\int }[\left(a\vec{H}+b\left(\vec{H}\cdot \hat{\underline{r}}\right)\hat{\underline{r}}\right)]G\left(\vec{r},\vec{r_{0}}\right)dV$$

where $\hat{\underline{r}}$ is the unit vector from the origin to the point source, $a=\mu_{r}\varepsilon_{r}-\frac{j\left(\mu_{r}-1\right)}{k_{0}R}\left(1-\frac{j}{k_{0}R}\right)$, $b=\left(\mu_{r}-1\right)\left(-1+\frac{3j}{k_{0}R}+\frac{3}{{\left(k_{0}R\right)}^{2}}\right)$, R denotes the distance between the source and field point, a and b are proportional to $1/R^{2}$(i.e., $k_{0}R\ll 1$). Hence $k_{0}^{2}a≅-\left(\mu_{r}-1\right)/R^{2}$ and $k_{0}^{2}b≅3\left(\mu_{r}-1\right)/R^{2}$.

The scattering signals from the region of interest with the negligible magnetic materials ($\mu_{r}=1$) and with the non-negligible magnetic materials ($\mu_{r}\neq 1$) can be rewritten as:

{(6a)H→scat(r0→)=−jωμ04π∫V(σ+jωε0εr′−σbg)H→G(r→,r0→)dV for (μr=1)(6b)H→scat(r0→)≅14π∫Vμr−1R2[−H→+3(H→·r_^)r_^)]G(r→,r0→)dV for (μr≠1)

The tissue conductivities are relatively small at low-frequency RF spectrum, such as 10 MHz. In this case, the Born approximation can be applied in the above equation when performing predictive forward modeling of a given object and coil configuration [31]. The induced field inside the object can be modeled approximately as the incident field that exists at the same location without place the object in the imaging region.

### 2.4. Image Processing 

Let us place the receiving sensor array at one of H vertical locations. The scattering signals from the target point P can be measured by each receiving sensor (see Figure 2). The visibility data of the scattering signals measured by a pair of receiving sensors can be calculated as [32]:

(7) $$\vec{V_{vi}}\left(\vec{r_{i}},\vec{r_{j}}\right)=\langle {\vec{H}}_{scat}\left(\vec{r_{i}}\right)\cdot {\vec{H}}_{scat}^{*}\left(\vec{r_{j}}\right)\rangle$$

where ${\vec{H}}_{scat}\left(\vec{r_{i}}\right)$ denotes the measured scattering field by receiving sensor located at $\vec{r_{i}}$, ${\vec{H}}_{scat}^{*}\left(\vec{r_{j}}\right)$ is the conjugate complex of measured scattering magnetic field by receiving sensor located at $\vec{r_{j}}$, <> denotes the time average. For a N-element receiving sensor array, the total visibility data is $\vec{V_{si}}=\overset{N}{\underset{i}{\sum }}\vec{V_{vi}}\left(\vec{r_{i}},\vec{r_{j}}\right)$, i≠j, N≥3, at a selected vertical height (the distance between the object and the receiving sensor array). This visibility data contains phase and amplitude information.

Define the object density as [33]:

(8) $$I\left(\vec{s}\right)={\left(\frac{j\omega \mu_{0}}{4\pi }\right)}^{2}{\left|\sigma \left(s\right)+j\omega \varepsilon_{0}\varepsilon_{r}-\sigma_{bg}\right|}^{2}\vec{H}\left(\vec{s}\right)\cdot \vec{H^{*}}\left(\vec{s}\right)$$

where $\sigma_{bg}$ is the conductivity of the medium.

The visibility formula over the object can be rewritten as:

(9) $$\vec{V_{vi}}\left(\vec{r_{i}},\vec{r_{j}}\right)={\left(\frac{j\omega \mu_{0}}{4\pi }\right)}^{2}\underset{V}{∭}{\left|\sigma \left(s\right)+j\omega \varepsilon_{0}\varepsilon_{r}-\sigma_{bg}\right|}^{2}\vec{H}\left(\vec{s}\right)\cdot \vec{H^{*}}\left(\vec{s}\right)\frac{e^{-jk_{0}\left(\vec{r_{i}}-\vec{r_{j}}\right)\cdot \underline{\hat{s}}}}{s^{2}}dV$$

Equation (11) can be simplified by combining Equations (8) and (9).

(10) $$\vec{V_{vi}}\left(\vec{D_{ij}}\right)=\underset{V}{∭}I\left(\vec{s}\right)\frac{e^{-j2\pi \vec{D_{ij}}\cdot \underline{\hat{s}}}}{s^{2}}dV$$

where $\vec{D_{ij}}$ is the baseline vector, $\vec{D_{ij}}=\left(\vec{r_{j}}-\vec{r_{i}}\right)/\lambda_{b}$ with $\lambda_{b}$ denotes the wavelength of background medium, $\underline{\hat{s}}=sin\theta cos\phi \underline{\hat{x}}+sin\theta sin\phi \underline{\hat{y}}+cos\theta \underline{\hat{z}}$, $dV=s^{2}sin\theta d\theta d\phi ds$. Change cartesian coordinates to spherical coordinates and define variables p=sinθcosϕ, q=sinθsinϕ, $n=cos\theta =\sqrt{1-p^{2}-q^{2}}$. Then, the element dV can be represented as:

(11) $$dV=s^{2}dpdqds/n$$

Substituting (11) into (10), visibility function becomes:

(12) $$\vec{V_{vi}}\left(\vec{D_{ij}}\right)=\underset{V}{∭}I\left(\vec{s}\right)\frac{e^{-j2\pi \vec{D_{ij}}\cdot \underline{\hat{s}}}}{n}dpdqds$$

where $\vec{D_{ij}}$ can be represented by $u_{ij}=\left(\vec{x_{j}}-\vec{x_{i}}\right)/\lambda_{b}$, $v_{ij}=\left(\vec{y_{j}}-\vec{y_{i}}\right)/\lambda_{b}$, $w_{ij}=\left(\vec{z_{j}}-\vec{z_{i}}\right)/\lambda_{b}$. If the coil array is positioned at a target vertical location, the visibility function can be expressed by the following equation:

(13) $$\vec{V_{vi}}\left(u_{ij},v_{ij},w_{ij}\right)=\underset{p}{\int }\underset{q}{\int }\underset{n}{\int }\frac{I\left(s,p,q\right)}{\sqrt{1-p^{2}-q^{2}}}e^{\left(-j2\pi \Phi_{ij}\right)}dpdqds$$

where $\Phi_{ij}=u_{ij}p+v_{ij}q+w_{ij}n$.

As all receiving sensors located on a planar plane ($w_{ij}=0$), the line integral can be obtained:

(14) $$\tilde{I}\left(p,q\right)=\underset{s}{\int }\frac{I\left(s,p,q\right)}{\sqrt{1-p^{2}-q^{2}}}ds$$

The visibility function changes to:

(15) $$\vec{V_{vi}}\left(u_{ij},v_{ij}\right)=\iint \tilde{I}\left(p,q\right)e^{-j2\pi \left(u_{ij}p+v_{ij}q\right)}dpdq$$

A 2D object image can be reconstructed by taking an inverse Fourier transform of Equation (15):

(16) $$\tilde{I}\left(p,q\right)=\iint \vec{V_{vi}}\left(u_{ij},v_{ij}\right)e^{j2\pi \left(u_{ij}p+v_{ij}q\right)}dudv$$

Equations (6), (7) and (16) can be applied to generate a 2D object image. The receiving sensor array plane is then placed at a new position in the Z-direction, and the 2D data collection process is repeated until the whole object has been scanned. Therefore, a 3D object image can be created by combining a stack of 2D HEI images. 

As shown in Figure 2b, the depth information can be expressed by:

(17) $$D_{n}=s_{n}cos\left(\theta_{n}\right)$$

where $\theta_{n}$ is the radiation or detection angle of the sensor. Then:

(18) $$dD=\frac{dz}{cos\left(\theta_{n}\right)}=\frac{dz}{\sqrt{1-p^{2}-q^{2}}}$$

A 3D object image can be reconstructed by combining all 2D images that obtained when the sensor array repositioned at different vertical locations:

(19) $$I\left(\vec{V_{vi}}=z_{n},p,q\right)=\frac{d\tilde{I}\left(p,q\right)\cdot \sqrt{1-p^{2}-q^{2}}}{dz}$$

The above equation can be represented as:

(20) $$\frac{d\tilde{I}}{dz}=\frac{{\tilde{I}}_{z_{n}}+{\tilde{I}}_{z_{n-1}}}{z_{n}+z_{n-1}}$$

A 3D image can be reconstructed by combining Equations (19) and (20). 

### 2.5. Metric 

The peak signal-to-noise ratio (*PSNR*) can be applied to serve as objective criteria:

(21) $$PSNR=10log_{10}\left(255/\sqrt{MSE}\right)$$

where *MSE* (mean squared error) measures the quality of an estimator, $MSE=\frac{1}{n}\overset{n}{\underset{i=1}{\sum }}{\left(Y_{i}-\hat{Y_{i}}\right)}^{2}$, Y is the vector of observed values of the variable being predicted, and $\hat{Y}$ is a vector of n predictions generated from a sample of n data points on all variables. *MSE* is always non-negative, and *MSE* closer to zero demonstrates better performance. 

The signal-to-noise ratio (*SNR*) measures the sensitivity.

(22) $$SNR=\mu_{sig}/\sigma_{bg}$$

where $\mu_{sig}$ and $\sigma_{bg}$ are the average signal value and the standard deviation of the background, respectively. 

The structural similarity index (*SSIM*) measures the image similarity:

(23) $$SSIM=\frac{\left(2\mu_{x}\mu_{y}+c_{1}\right)\left(2\sigma_{xy}+c_{2}\right)}{\left(\mu_{x}^{2}+\mu_{y}^{2}+c_{1}\right)\left(\sigma_{x}^{2}+\sigma_{y}^{2}+c_{2}\right)}$$

where $\mu_{x}$ and $\mu_{y}$ are the average of the original image x and reconstructed image y, respectively. $\sigma_{x}^{2}$ and $\sigma_{y}^{2}$ are the variance of x and y, $\sigma_{xy}$ is the covariance of x and y. The two variables $c_{1}={\left(0.01L\right)}^{2}$ and $c_{2}={\left(0.03L\right)}^{2}$ to stabilize the division with the weak denominator, L is the dynamic range of pixel-values. Where −1≤SSIM≤1, and SSIM=1 can be obtained when x=y.

## 3. Numerical Experiments

A numerical system, including a realistic head model, a 16-element coil array, and imaging process model, was developed to evaluate the effectiveness, sensitivity, and accuracy of the proposed method for imaging infarcts that mimic a stroke embedded in the realistic head model. All simulations were conducted using MATLAB 2018a (The MathWorks, Inc. Natick, MA, USA) on a Dell Precision 5820 workstation which has an Intel Xeon W-2145 CPU with an internal clock frequency of 3.7 GHz and 256 GB of memory. 

### 3.1. Simulation Setup

**Figure 3:**
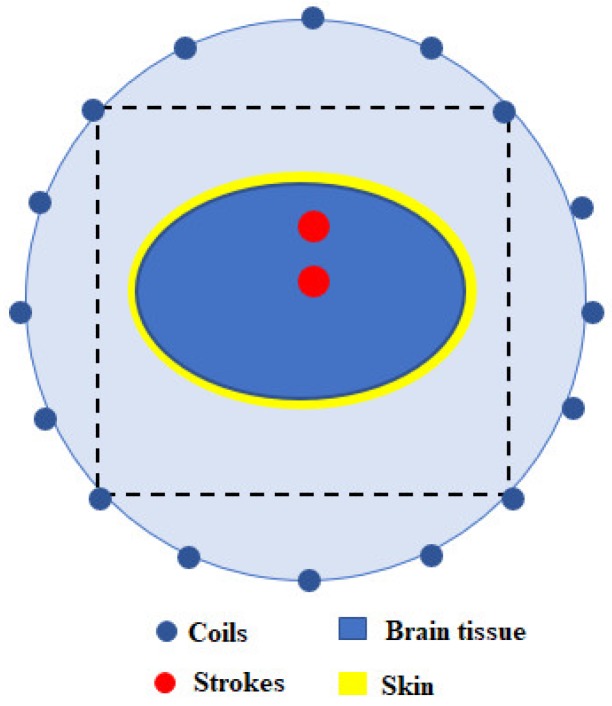
Head model and receiver array configuration.

Figure 3 illustrates the top view of the numerical system setup. The system consisted of 16 excitation coils and 16 receiving coils mounted on the wall of a cylindrical tank (80 mm in radius, 100 mm in height). All excitation coils were equally arranged in a circle, which positioned under the cylindrical tank. All receiving coils were equally arranged in a circular array, and this array plane was designed moveable in the Z-direction. A 3D head phantom was placed at the center of the cylindrical tank (x = 0 mm, y = 0 mm, z = 0 mm). A high dielectric medium ($\varepsilon_{r}=88$, σ=0.48 S/m, similar to distilled water) was filled in the imaging chamber (cylindrical tank), and a single frequency of 10 MHz was selected as working frequency based on previous studies [26,29]. To collect 3D image data, the head model was placed at z = 0 mm, the sensor array place was moved from axial z = −50 mm to z = 50 mm in steps of 1 mm. For each vertical location, a 2D image data set was recorded to produce a 2D image. This data collection process was repeated until the entire head model has been scanned in Z-direction and a total number of 3,840,000 3D image data was collected. A 3D object image was reconstructed by combining a stack of 2D images.

### 3.2. RF Coil

For simplicity, the single circle loop coil (radius of 25 mm) was simulated as excitation sensor and receiver [29]. The magnetic field induced by each excitation coil can be computed by using Biot-Savart law:

(24) $$\vec{H}=\frac{\mu_{0}I}{4\pi r_{c}{}^{2}}\oint dL=\frac{\mu_{0}I}{4\pi r_{c}{}^{2}}2\pi r_{c}=\frac{\mu_{0}I}{2r_{c}}$$

where I is the current in excitation coil, $r_{c}$ denotes the radius of the loop coil, and L denotes the length of the coil. The current in the coil was 1 A. 

### 3.3. Head Model

An ellipsoidal shaped head model (long radius 60 mm, short radius 55 mm, thickness radius 40 mm) was developed to provide the validity of the proposed method. The head model consisted of a skin layer (3 mm thick), fat (5 mm thick), skull (7 mm thick), cerebro-spinal fluid (CSF, 3 mm thick), grey matter, white matter, and dura. Sphere-shaped inclusion was inserted into the head phantom to simulate infarcts that mimic a stroke. The brain lesion was assumed to consist of 100% blood. In this study, two inclusions (different sizes and positions) have been investigated. We used the published EPs values of the tissues to develop the head model [34]. Table 1 shows EPs of tissues at a frequency of 10 MHz. To save the computational time, the head model contained 80×80×50 voxels which correspond to a region of 160×160×100 mm^3^. The EPs of tissues have been assigned for each voxel to develop head phantom. The Gaussian function was applied to demonstrate the head model and brain lesion model.

## 4. Results 

**Figure 4:**
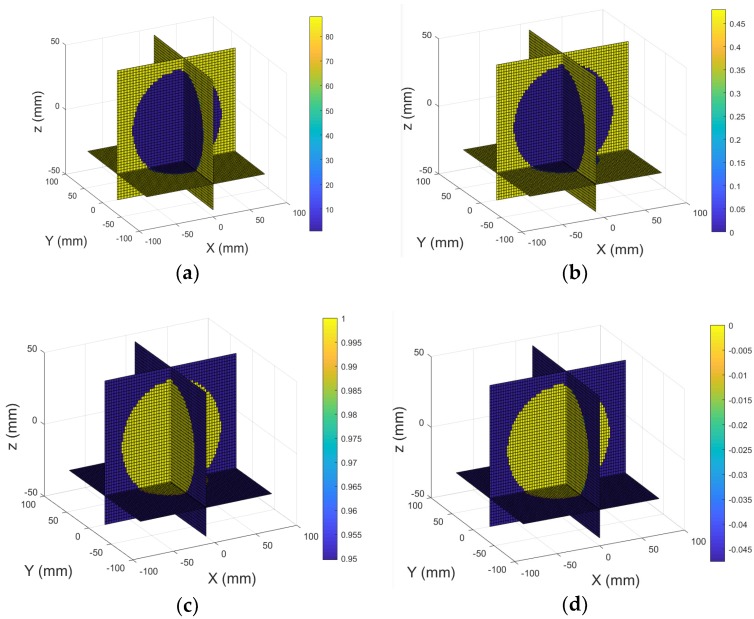
3D view of the heal model contains unit relative permittivity: (**a**) Real part; (**b**) Imagery part; 3D reconstructed images: (**c**) Real part; (**d**) Imagery part.

Several simulations have been performed to test the detectability of inclusions embedded in the multilayer head model using the proposed method. The first simulation performance was carried out with the conductivity set to zero and the relative permittivity set to unity ($\varepsilon_{r}=$ 1) for all tissues, thus simulating the host medium. Figure 4a,b display the real part (relative permittivity) and imagery part (conductivity) of the model under test, and its 3D reconstructed images are shown in Figure 4c,d. 

**Figure 5:**
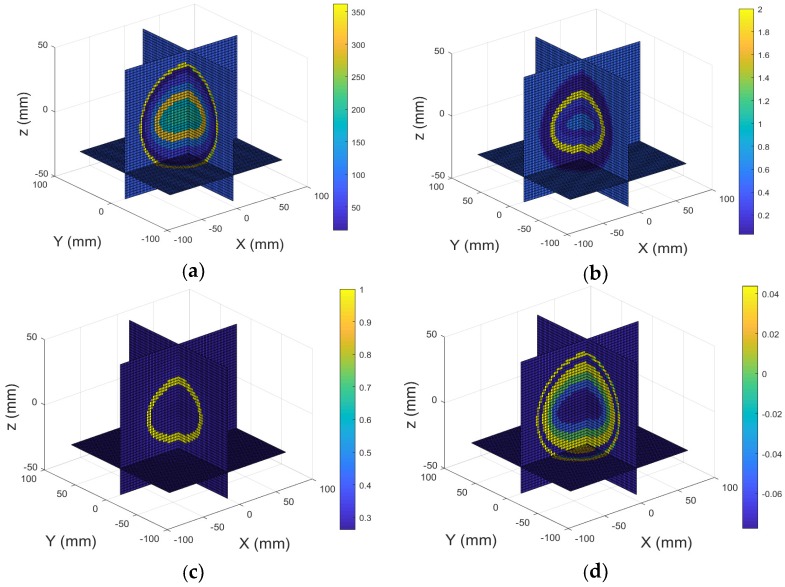
3D view of the head model with no brain lesion: (**a**) Real part; (**b**) Imagery part; 3D reconstructed images of the head model with no brain lesion: (**c**) Real part; (**d**) Imagery part.

The second simulation experiment was conducted with the conductivity and relative permittivity values set to the correct information (see Table 1), with no brain lesion present. Figure 5a,b show the real part (relative permittivity) and imagery part (conductivity) of the head model with no brain lesion and its 3D reconstructed images are demonstrated in Figure 5c,d. Results show that the internal structures of the head model can be clearly identified in the imagery part of the 3D reconstructed image. However, not all internal structures of the head model can be observed in the real part of the 3D reconstructed image. The computational time was 303.98 seconds for the first two simulation experiments.

**Figure 6:**
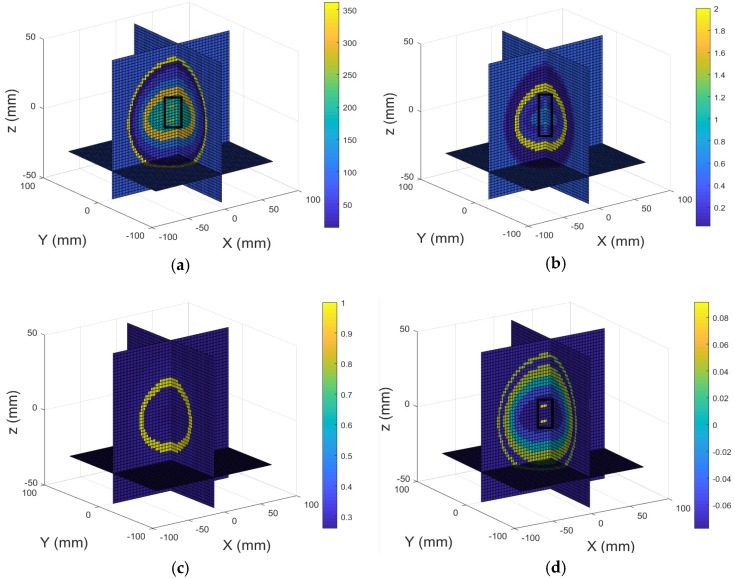
3D view of the head model contains two lesions: (**a**) Real part; (**b**) Imagery part; 3D reconstructed images of the head model contains two lesions: (**c**) Real part; (**d**) Imagery part.

The third experiment was carried out with the head model contains two lesions present in the imaging chamber. As shown in Figure 6a,b, the head model under test contains two lesions where brain lesion A (5 mm in radius, squared in black) located at (0 mm, 0 mm, 8 mm) and brain lesion B (5 mm in radius, squared in black) located at (0 mm, 0 mm, −8 mm). The real part and imaginary part of the 3D reconstructed images are shown in Figure 6c,d. Results show that two inclusions can be clearly identified in the imagery part of the 3D reconstructed image with the correct size, shape, and location information. However, no inclusion can be observed in the real part of the reconstructed image. The total computational time for this simulation experiment was 297.232 s.

**Figure 7:**
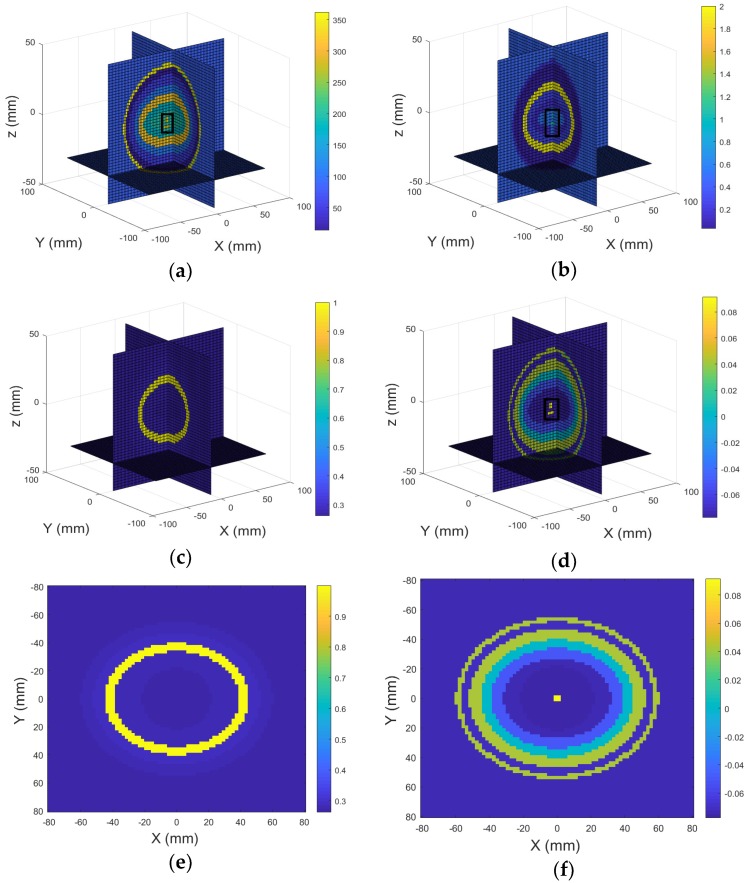
3D view of the head model contains two lesions: (**a**) Real part; (**b**) Imagery part; 3D reconstructed images of the head model contains two lesions: (**c**) Real part; (**d**) Imagery part; 2D reconstructed image: (**e**) Real part; (**f**) Imagery part.

The fourth experiment was carried out with the head model contains two different size lesions present in the imaging chamber. As shown in Figure 7a,b, the head model under test contains two lesions, brain lesion A (3 mm in radius, squared in black) located at (0 mm, 0 mm, 0 mm) and brain lesion B (5 mm in radius, squared in black) located at (0 mm, 0 mm, −8 mm). The real part and imaginary part of the 3D reconstructed images are shown in Figure 7c,d. Results show that two inclusions can be clearly identified in the imaginary part of the 3D reconstructed image with the correct size, shape, and location information. However, no inclusion can be observed in the real part of the reconstructed image. The total computational time for this simulation experiment was 297.232 s. 

We have compared the proposed method with the developed 2D HEI method. To collect 2D image data, we have placed the sensor array plane and the head model at z = −50 mm and z = 0 mm, respectively. Figure 7e,f present the real part and imagery part of the 2D reconstructed images (XY plane) of the head model, respectively. However, only one of two inclusions can be observed in the imagery part of the 2D reconstrued image. The total computational time to produce the 2D images was 157 s.

**Figure 8:**
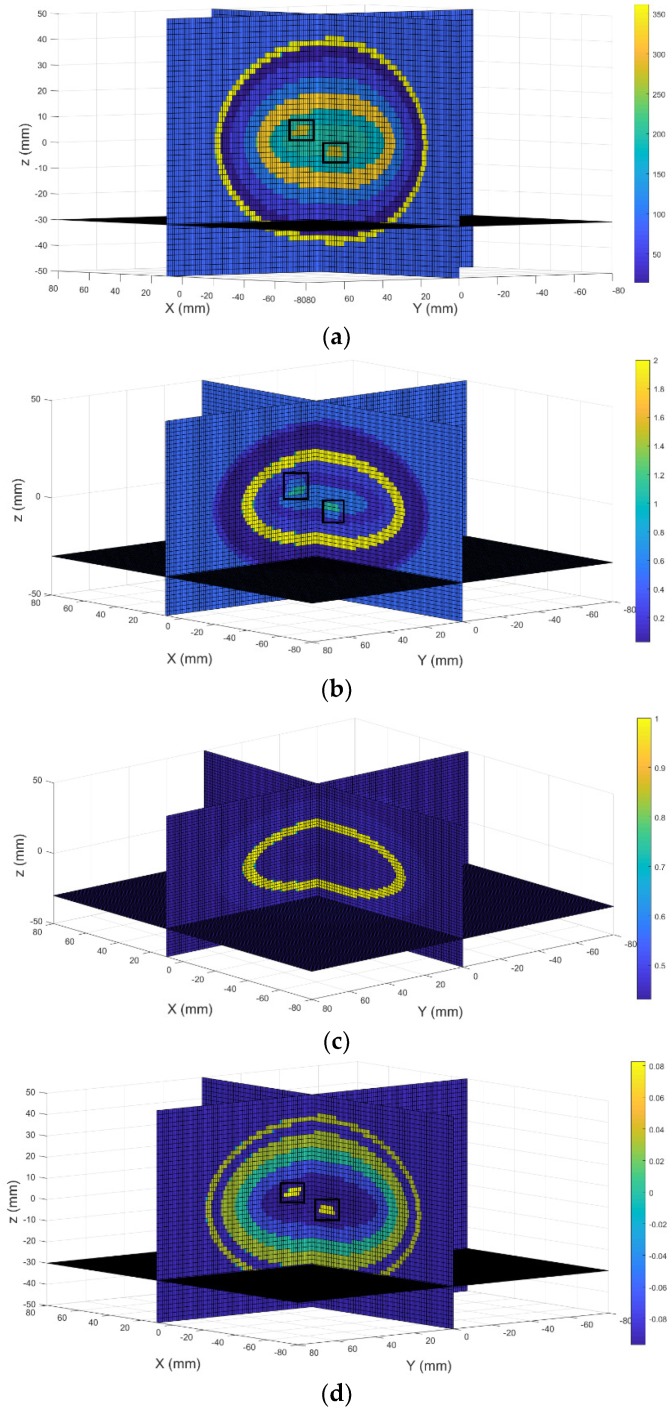

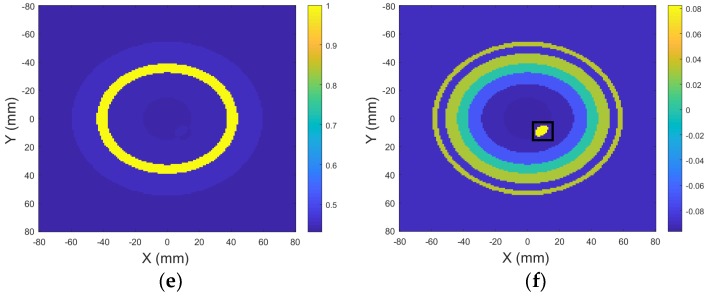
3D view of the head mode: (**a**) Real part; (**b**) Imagery part; 3D reconstructed images: (**c**) Real part; (**d**) Imagery part; 2D reconstructed images: (**e**) Real part; (**f**) Imagery part.

The fifth simulation was conducted using the head model contains two inclusions (5 mm in radius, squared in black) located at (10 mm, 10 mm, 6 mm) and (0 mm, 0 mm, −6 mm). Figure 8a,b show the permittivity and conductivity values of the head model. Figure 8c,d display the real part and imaginary part of 3D reconstructed images, respectively. Figure 8e,f present the real part and imaginary part of the 2D reconstructed images, respectively. Results show that two inclusions can be observed in the imaginary part of the 3D reconstrued image with the correct size, shape, and location information. However, only one of two inclusions can be observed in the imagery part of the 2D reconstrued image.

Color bars in the original images plot the permittivity and conductivity values of the model under test, while color bars in the constructed images demonstrate the backscattered energy density distributions in the reconstructed images. Not all internal structures of the brain tissues can be identified in the reconstructed images especially the real part (permittivity) of the reconstructed images. The conductivity reconstructions (imaginary part) have higher quality than the permittivity reconstructions (real part), this finding agrees with the previously published research outcomes [35,36]. A total number of (16×16×15=3840) 2D image data, and (16×16×15×100=384,000) 3D image data were measured to produce a 2D image and a 3D image using the proposed method. Thus, more helpful information can be obtained in the reconstructed 3D images compared to the 2D images. Table 2 shows simulation errors. It can be seen that with the proposed method it is much easier to identify larger size inclusions. Compared to previous published 2D image results [29], the proposed method could detect two inclusions which were located at the same XY plane but in different YZ planes.

## 5. Conclusions

This paper reported the theoretical framework of 3D HEI for internal structure imaging of small inclusions embedded in biological tissue. A numerical system, including a realistic 3D head model, a 16-element excitation sensor array, and a moveable 16-element receiving sensor array as well as image processing model, has been developed to test the detectability of lesion using the proposed method. Several simulation experiments have been conducted to evaluate the effectiveness, accuracy, and sensitivity of the proposed framework for detecting brain lesions embedded in the head model. A comparison study between the 2D HEI and 3D HEI has been conducted through various simulation experiments. The results showed that the 3D HEI could produce 3D brain images and detect inclusions with the correct size, shape, and location information. The two inclusions could be successfully observed in the reconstructed 3D images even when they were located at the same XY plane but in different YZ planes. 

The HEI image quality highly depends on the visibility data that compares the scattering signals measured by any pair of receivers. For 16 excitation sensors and a 16-element receiver array that placed at 100 different vertical locations, a total number of [16×16×(16−1)×100=384,000] data can be recorded to produce a 3D image using the proposed method, while only a total number of (16×16=256) data can be measured to produce a 2D image using the conventional MIT method. Thus, the proposed method has the potential to produce a higher resolution image compared to the conventional EM techniques. Previous experimental studies [37] have shown that the 2D image quality also depends on the sensor array configuration and the sensor number. However, whether this claim is suitable for the practical 3D HEI system is one of the target future works. 

Head is a very complex organ with functions and mechanisms have not been fully discovered. EMI technique exploits structural data of different levels, such as EPs of brain tissues, will help one to understand the internal structure of brain tissues, which offers the potential for detecting and monitoring brain disease. However, only ideal models (noise free) were involved in this study; this may affect the effectiveness of the proposed method. The image quality could be degraded due to the noise acquired from the practical measurement instrument. Furthermore, the proposed method requires further validation in more realistic experiment scenarios. From an experimental point-of-view, develop an EM device to collect complete EM data for biomedical applications remains both highly desirable and extremely challenging.

The obtained theoretical results showed that the proposed framework has the potential to become a useful tool for realizing complex situations and optimizing the whole system before the device is tested in practice. Future work should evaluate more realistic experimental data gathered from physical models, biological tissues, animals, and human subjects, to ensure that the proposed method is robust to more realistic scenarios. The development of a sensitive sensor for practical implementation of the proposed method is another target future research direction.

## Figures and Tables

**Table 1:** EPs of tissues at 10 MHz [34].

Structure	Thickness (mm)	*σ* (S/m)	Relative Permittivity
Matching medium	20	0.48	88
Skin	3	0.1973	361.66
Fat	5	0.029	13.767
Skull	7	0.0828	53.8
CSF	3	2	109
Grey matter	6	0.292	320
White matter	7	0.159	176
Dura	5	0.544	194.93
Blood	3/5	1.097	280

**Table 2:** Simulation errors.

Model	Head Contains Two Inclusions (5 mm in Radius) Located at (0 mm, 0 mm, 8 mm) and (0 mm, 0 mm, −8 mm)	Head Contains Two Inclusions (3 mm, 5 mm in Radius) Located at (0 mm, 0 mm, 0 mm) and (0 mm, 0 mm, −8 mm).	Head Contains Two Inclusions (5 mm in radius) Located at (10 mm, 10 mm, 6 mm) and (0 mm, 0 mm, −6 mm)
PSNR (dB)	49.9943	36.6764	49.9943
SNR (dB)	5.6384	2.0651	5.6382
MSE of real part	0.9657	0.9657	0.9657
MSE of Imagery part	0.4896	0.0876	0.0498
SSIM of real part	0.0455	0.4896	0.04986
SSIM of Imagery part	0.0455	0.0455	0.0455
